# Impact of Varying Dosages of Fish Oil on Recovery and Soreness Following Eccentric Exercise

**DOI:** 10.3390/nu12082246

**Published:** 2020-07-27

**Authors:** Trisha A. VanDusseldorp, Kurt A. Escobar, Kelly E. Johnson, Matthew T. Stratton, Terence Moriarty, Chad M. Kerksick, Gerald T. Mangine, Alyssa J. Holmes, Matthew Lee, Marvin R. Endito, Christine M. Mermier

**Affiliations:** 1 Department of Exercise Science and Sport Management, Kennesaw State University, Kennesaw, GA 30144, USA; gmangine@kennesaw.edu (G.T.M.); alyssajh@hotmail.com (A.J.H.); mlee120@students.kennesaw.edu (M.L.); 2 Department of Kinesiology, California State University Long Beach, Long Beach, CA 90840, USA; kurt.escobar@csulb.edu; 3 Department of Exercise and Sport Science, Coastal Carolina University, Conway, SC 29528, USA; kjohns10@coastal.edu; 4 Kinesiology and Sport Management, Texas Tech University, Lubbock, TX 79409, USA; matthew.stratton@ttu.edu; 5 Department of Kinesiology, University of Northern Iowa, Cedar Falls, IA, 50614, USA; terence.moriarty@uni.edu; 6 School of Health Sciences, Lindenwood University, St. Charles, MO 63301, USA; ckerksick@lindenwood.edu; 7 Department of Health, Exercise and Sports Sciences, University of New Mexico, Albuquerque, NM 87131, USA; ray87323@yahoo.com (M.R.E.); cmermier@unm.edu (C.M.M.)

**Keywords:** muscle damage, muscle recovery, omega-3 polyunsaturated fatty acids, fish oil, eccentric exercise

## Abstract

Fish oils (FOs) are rich in omega-3 long-chain polyunsaturated fatty acids, which have been purported to enhance recovery of muscular performance and reduce soreness post-exercise. However, the most effective FO dose for optimizing recovery remains unclear. The purpose of this investigation was to examine the effect of FO supplementation dosing on the recovery of measures of muscular performance, perceived soreness, and markers of muscle damage following a rigorous bout of eccentric exercise. Thirty-two college-aged resistance-trained males (~23.6 years, 71.6 kg, 172.1 cm) were supplemented with 2, 4, 6 g/day (G) FO or placebo (PL) for ~7.5 weeks. Following 7 weeks of supplementation, pre-exercise (PRE) performance assessments of vertical jump (VJ), knee extensor strength, 40-yard sprint, T-test agility, and perceived soreness were completed prior to a bout of muscle-damaging exercise and were repeated immediately post (IP), 1-, 2-, 4-, 24-, 48-, and 72-h (H) post-exercise. Repeated measures analysis of variance indicated a treatment × time interaction (*p* < 0.001) for VJ and perceived soreness, but no group differences were observed at any time point. VJ returned to PRE (54.8 ± 7.9 cm) by 1H (51.8 ± 6.5 cm, *p* = 0.112) for 6G, while no other groups returned to baseline until 48H. Lower soreness scores were observed in 6G compared to PL at 2H (mean difference [MD] = 2.74, *p* = 0.046), at 24H (MD: 3.45, *p* < 0.001), at 48H (MD = 4.45, *p* < 0.001), and at 72H (MD = 3.00, *p* = 0.003). Supplementation with 6G of FO optimized the recovery of jump performance and muscle soreness following a damaging bout of exercise.

## 1. Introduction

It is well known that exercise induces both mechanical and metabolic stress [1,2,3,4,5]. While those who are accustomed to exercise will often recover from an exercise session with limited negative repercussions, those engaging in unaccustomed exercise or rigorous training (e.g., eccentric exercise) often experience undesirable side effects. These may include impaired muscle function [6,7], such as decreases in strength, range of motion, and soreness [8,9]. Such outcomes are often products of immediate post-exercise morphological alterations in the activated musculature [10], as well as increases in inflammation [11] and markers of oxidative damage [12,13]. Such physiological alterations may delay recovery and decrease subsequent performance.

To facilitate faster recovery and maintain subsequent training session volumes, intensity, and performance, individuals who exercise and train employ a range of recovery strategies [14], including massage therapy [15], myofascial release [16], stretching [17], nonsteroidal anti-inflammatories [18], compression garments [19], and cryotherapy/cryostimulation [20], among others. While these recovery strategies have been shown to be successful in alleviating some of the symptoms of skeletal muscle damage, nutritional strategies have also been proposed to mitigate the negative effects that one may experience following a damaging/rigorous bout of exercise [21]. One dietary strategy that has garnered interest is consumption of omega-3 (ω-3) long-chain polyunsaturated fatty acids (LCPUFAs) [22]. The discovery of ω-3 LCPUFA’s pleiotropic effects on human health (e.g., cardioprotective [23,24,25], triglyceride lowering [26,27,28], and anti-inflammatory [29,30,31]) have driven significant medical and public interest, but of late, ω-3 LCPUFAs have caught the exercise community’s attention. ω-3 LCPUFAs, hereinafter referred to as ω-3, are commonly obtained via the diet by consuming oily fish, such as tuna and salmon, and may also be obtained via oral supplementation, such as fish oil (FO) soft-gel supplements.

Interest in the impact of the ω-3 on “recovery”, or more specifically eicosapentaenoic acid (EPA) and docosahexaenoic acid (DHA) in the form of FO supplementation, have been investigated using an array of interventions, spanning damaging eccentric contractions [32,33,34,35], exhaustive endurance exercise [36], and immobilization [37], in trained and untrained men and women [37,38,39,40], with only limited work completed in women [35,40,41,42]. Current data suggests, and as highlighted in a comprehensive systematic review by Heileson and Funderburk (2020), that FO supplementation likely enhances recovery and preserves strength following stressful exercise. More specifically, and as it relates to this literature, the term recovery may be defined as positive modulation in blood biomarkers associated with muscle damage and fatigue, reductions in muscle swelling or soreness/pain, improvements in joints’ range of motion, and improvements, maintenance, or a decrease in the reduction of muscular performance in the recovery period, such as jump height or muscular strength. The positive influence of ω-3 on these outcome measures has mostly been attributed to the anti-inflammatory and immunomodulatory properties conferred by these fatty acids [43].

To date, conflicting results are present regarding FO’s ability to enhance recovery, which may be partially attributed to the varying dosages of FO implemented (i.e., 1.8–6.0 g/day), as well as the length of supplementation. While data suggest several positive impacts of EPA and DHA obtained from FO supplements on recovery, there has yet to be a consensus on the dosage. Further, to the best of our knowledge, no study has compared different dosages of FO supplements on recovery from very strenuous muscle-damaging exercise. Thus, a FO dose–response investigation is warranted and needed. It has been suggested that the minimum effective dose to enhance recovery is a 2G FO dose for at least four weeks [22], but more research is needed to fully examine this recommendation. Therefore, we sought to contribute to the gap in the scientific literature by investigating the dose–response of FO supplementation on recovery from damaging resistance exercise. More specifically, the purpose of the investigation was to examine the effect of 7 weeks of 2G, 4G, and 6G of FO supplementation on markers of recovery (muscular performance, perceived muscle soreness, markers of muscle damage) following damaging eccentric exercise in both men and women.

## 2. Materials and Methods

### 2.1. Experimental Approach

**Figure 1:**
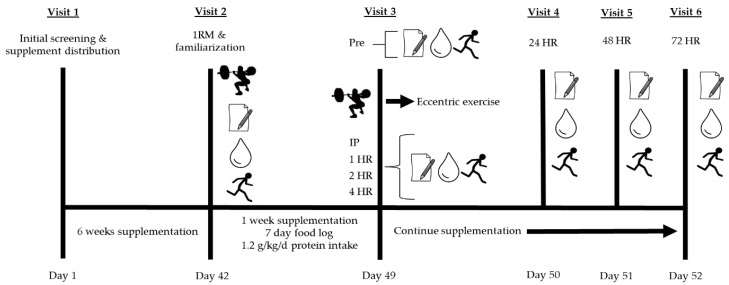
Study design overview. Participants (n = 32) were randomly assigned to consume 2, 4, or 6 g/day fish oil or placebo. 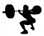 = damaging bout of eccentric exercise; 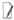 = perceived muscle soreness; 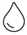 = blood collection for assessment of creatine kinase and lactate dehydrogenase; 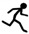 = muscular performance (vertical jump, 40-yard dash, T-test agility, maximal voluntary isometric contraction).

Utilizing a randomized placebo-controlled double-blind experimental design, participants were randomly assigned to consume 2- (2G), 4- (4G), or 6- (6G) g/da of either FO or placebo (PL) supplementation for ~7.5 weeks (8 participants per group (4 males and 4 females per group); a 6-week run in the supplementation period, 1 week involving familiarization testing at the beginning of the week and experimental testing at the end of the week, and three days of recovery testing). Muscle soreness, venous blood (for the assessment of creatine kinase (CK) and lactate dehydrogenase (LDH), and indices of muscle function were collected prior to eccentric exercise, as well as immediately post, 1-, 2-, 4-, 24-, 48-, and 72-h (H) post-exercise. Participants continued to supplement until they completed the 72H time-point. A study overview can be found in Figure 1. The study was approved by the University of New Mexico (UNM) Institutional Review Board (HRRC #15–192) and data were collected in accordance with the Declaration of Helsinki.

### 2.2. Participants

All participants were informed verbally as to the aims and risks of the study as well as provided written informed consent prior to study enrollment and participation. A total of 41 men and women were recruited for this research study. Participants were recruited via flyers, email announcements, and word of mouth. Sixteen males (n = 16; 23.8 ± 2.7 years 81.5 ± 9.9 kg, 175.7 ± 4.5 cm) and 16 females (n = 16; 23.4 ± 3.1 years, 61.7 ± 7.2 kg, 170.4 ± 6.2 cm) completed the study (Table 1). Four males and four females made up each supplement group. Participants were defined as recreationally active: Currently engaging in resistance exercise, 3–5 days per week, with a minimum of 3-H per week and a maximum of 8-H per week and no more than 2-H of aerobic exercise per week. All individuals were apparently healthy without a history of disease or current medication use. Participants were screened for supplementation use and only enrolled if they had not consumed fish oil supplements one-year prior, creatine two months prior, and beta-alanine one month prior to study enrollment. They were permitted to consume protein supplements and multivitamins during the 6-week run in the supplementation period but asked to discontinue for the final 1.5-weeks of the investigation. Participants who consumed 2 servings or greater of fish per week on a consistent basis were also excluded. Females were excluded if they had taken any form of hormonal contraception one year prior to participation in the study. Of the nine participants who dropped out of the study, five (2 females, 3 males) removed themselves due to scheduling conflicts and four were removed for missing 5 or more days of supplementation (1 female and 4 males). Regarding dropouts from randomly assigned groups, the placebo, 2G, 4G, and 6G groups each experienced dropouts; specifically, two, two, three, and two individuals dropped out from their assigned groups, respectively. No adverse events were reported by any of the participants at any time related to supplementation.

### 2.3. Visit 1: Initial Screening and Supplement Distribution

Following written informed consent and determination of study involvement, participants’ height and body mass were assessed using a calibrated scale (Tanita Model #3101, Arlington Heights, IL, USA) and stadiometer (Seca, Chino, CA, USA). Following this, the 3-site skinfold (Lange Skinfold Caliper, Cambridge Scientific Industries, Cambridge, MD, USA) measurement was collected and body fat percentage was determined using the sex-specific Jackson–Pollock 3-site skinfold technique [44,45] for demographic purposes. All participants were then counseled on how to complete dietary food logs, dietary recommendations, and physical activity logs, and were randomly assigned to a supplement group, given their supplements, and then scheduled to return at the end of their 6-week supplementation regimen (day 42) (Figure 1).

#### Supplementation and Diet

Participants ingested either 2G, 4G, or 6G of either FO (MusclePharm, MusclePharm, Denver, CO, USA) or placebo (PL) (safflower oil, MusclePharm) daily for ~7.5 weeks following visit 1. Each FO capsule contained 400 mg of EPA and 300 mg of DHA, and provided a total FO dose of 1000 mg. Table 2 shows the EPA and DHA breakdown for each FO supplement group (divided into two doses per day—morning and evening). Regardless of group, pills were the same shape, color, size, and texture. Supplementation compliance was defined as consuming the assigned supplement on 47 of the 52 required days (~90%) and no missed supplements during the final 1.5-weeks of testing. Compliance was checked by a member of the research team on a bi-weekly basis.

Participants were asked to maintain their normal dietary intake up until one week before pre-testing (or day 49), with which a protein intake of 1.2 g/kg/day was recommended throughout the remainder of the study. A registered dietician was made available to counsel individuals on protein intake, if needed.

### 2.4. Visit 2: One-Repetition Maximum (1RM), Familiarization, and Diet Counseling

Following 6-weeks of supplementation, participants returned to the laboratory for one-repetition maximum (1RM) and familiarization of muscle function indices. Participants were asked to bring their dietary recall sheets (2 weekdays and 1 weekend day) for the past 6-weeks, as well as make note of any changes to their training habits. A member of the research team also made a note of supplement dosages missed, if applicable. Back squat 1RM was determined according to methods previously described (Kraemer et al. 1995) using a Smith machine (Pro-Elite Strength Systems, Salt Lake City, UT, USA). Following a 10-min self-selected dynamic warm-up, each participant completed a specific warm-up that included one set of 8–10 repetitions at 50% of their estimated (est) 1RM), a second set of 3–5 repetitions at ∼75% of 1RM_est_, and a third set of 1–3 repetitions at ∼90% of 1RM_est_. After the warm-up, each participant’s 1RM was determined within 3 maximal one-repetition trials separated by 3–5 min of rest. Attempts was defined as successful when the participant reached a depth equal to 90° of knee flexion and returned to their starting position (i.e., knees and hips at full extension). Spotters were present to provide verbal encouragement and spotting to ensure the safety of the subjects.

Following the 1RM assessment, participants were asked to rest for 10 min, and then completed a thorough familiarization of the countermovement vertical jump (VJ), T-test agility, 40-yard (yd) sprint, and maximal voluntary isometric contraction (MVIC) of the quadriceps at 120° of knee flexion to eliminate any learning effects on test performance during data collection. During the 10 min of rest, participants were familiarized with the visual analog scale used for the assessment of perceived muscle soreness. Following the conclusion of the 1RM assessment and familiarization of performance assessments, participants were counseled on maintaining their normal caloric intake, while adhering to a protein intake of 1.2 g/kg/day throughout the remainder of the study. A registered dietician was available for counsel if necessary. Participants were asked to return one week later to complete the investigational protocol.

### 2.5. Visit 3: Investigational Protocol (Muscle-Damaging Exercise Visit)

Upon arrival to the laboratory, a phlebotomist collected the first (pre-exercise) of five blood samples, followed by pre-exercise assessment (PRE) of perceived soreness, VJ, T-test agility, 40-yd sprint, and MVIC. Participants then completed a 10-min self-selected dynamic warm-up followed by the muscle-damaging squat exercise protocol. Following completion of the exercise protocol, participants had their blood drawn, rated their current perceived muscle soreness, and completed all measures of muscle performance testing, IP, 1-, 2-, 4-, 24-, 48-, and 72-H post-squat exercise.

#### Resistance Exercise Protocol

Participants completed eccentric squats (4-s lowering phase and 1-s upward phase) in order to induce muscle damage. All participants completed the squat exercise: 10 sets of 8 repetitions, 70% 1RM using a Smith machine. Three minutes of rest was permitted between sets. Following the 10th squat set, participants completed 5 sets of 20 consecutive split jump-squats using only their body weight. Two minutes of rest was permitted between sets. Following competition of set 5, the muscle-damaging resistance exercise protocol was considered complete.

### 2.6. Blood Collection, Perceived Soreness, and Muscle Performance Indices

#### 2.6.1. Blood Collection and Assessments

Blood was collected pre-muscle-damaging exercise, IP, 1-, 2-, 4-, 24-, 48-, and 72-H post-exercise for the collection of plasma. Blood samples were collected from the antecubital vein, centrifuged at 1650× *g* for 10 min, and stored at −80 °C until analysis. As indirect markers of muscle damage, plasma concentrations of CK and LDH were determined at pre-muscle-damaging exercise, 4-, 24-, 48-, and 72-H. CK (Pointe Scientific, Canton, MI, USA) and LDH (Pars Azmoon Inc. Tehran, Iran) were determined in duplicate using enzymatic assays according to the manufacturer’s guidelines.

#### 2.6.2. Perceived Soreness

A paper-version visual analog scale was used to assess perceived soreness. Zero centimeters represented no soreness, while 10 cm represented extreme soreness. Participants rated their perceived soreness at all time-points (pre-exercise, IP, 1-, 2-, 4-, 24-, 48-, and 72-H).

#### 2.6.3. Vertical Jump

Following appropriate determination of their standing height, the participant’s maximum countermovement VJ was assessed using a Vertec device (Perform Better, West Warwick, RI, USA). Participants were allowed three jumps, with the highest jump recorded and used for statistical analysis. Two minutes of rest was given between attempts.

#### 2.6.4. T-Test Agility Test

The T-test is used to measure agility. Participants began with both feet behind the tape line. Participants were instructed to sprint forward (from the start cone) (10-yd) and touch a cone directly in front of them, then lateral shuffle to the left (5-yd) and touch a second cone, then shuffle right (10 yd) and touch a third cone, before shuffling back, left, 5-yd to the middle, and backpedaling past the start cone (5-yd) to finish the test. Two stopwatch timers (Pro survivor, 6013-3v, AccuSplit, Pleasanton, CA) were used to measure the total time in seconds(s) from the start of the movement until the participant crossed the finish line. An average of the two timers’ times was used for statistical analysis.

#### 2.6.5. Forty-Yard Sprint

Participants were asked to sprint 40-yd as fast as possible in a straight line down a non-occupied Terazzo Tile surface hallway. They were instructed to begin with their preferred foot forward and placed on a line taped on the floor from a standing position. The same forward foot was used for all test timepoints. Participants performed three sprints and an automatic digital timer linked to sensors (Bower Timing Systems, Draper, UT) was placed at the 0- and 40-yd marks to assess the total sprint time.

#### 2.6.6. Maximal Voluntary Isometric Contraction

Using the Biodex System 4 (Shirley, NY, USA), participants’ MVIC strength of the dominant limb knee extensor was assessed. Each participant’s Biodex chair position was kept standard for all visits and attempts. The participant’s knee was placed at 120° of flexion. Three 5-s contractions were completed with one minute of rest between attempts. The attempt resulting in the highest peak torque (newton-meters) was recorded and used for statistical analysis.

### 2.7. Statistical Analysis

A one-way analysis of variance (ANOVA) was performed to determine between-group differences at baseline. To determine the effect of FO dosing, separate treatment (4 levels: PL, 2G, 4 G, 6G) × time (8 levels: PRE, and IP, H, 2H, 4H, 24H, 48H, and 72H post-muscle-damaging exercise) ANOVA with repeated measures was completed on all performance measures (i.e., vertical jump height, T-test, 40-yd sprint, and MVIC), CK, LDH, and perceived muscle soreness data. In the event of a significant F-ratio, a repeated measures ANOVA with a Bonferonni correction was performed on each group separately to determine the main effect of time and specific time differences. Additionally, separate one-way ANOVAs were performed at IP, 1H, 2H, 4H, 24H, 48H, and 72H to examine group differences at each time point followed by Tukey’s honestly significant difference post-hoc analysis with any significant F-ratio. All between-group differences were further analyzed using effect sizes (η^2^: Partial eta squared). Interpretations of the effect size were evaluated [46] at the following levels: Small effect (0.01–0.058), medium effect (0.059–0.137), and large effect (>0.138). A criterion alpha level of *p* ≤ 0.05 was used to determine statistical significance. All data are reported as mean ± standard deviation. SPSS statistical software (V. 24.0, Chicago, IL, USA) was used for all analyses.

## 3. Results

### 3.1. Performance Measures

**Figure 2:**
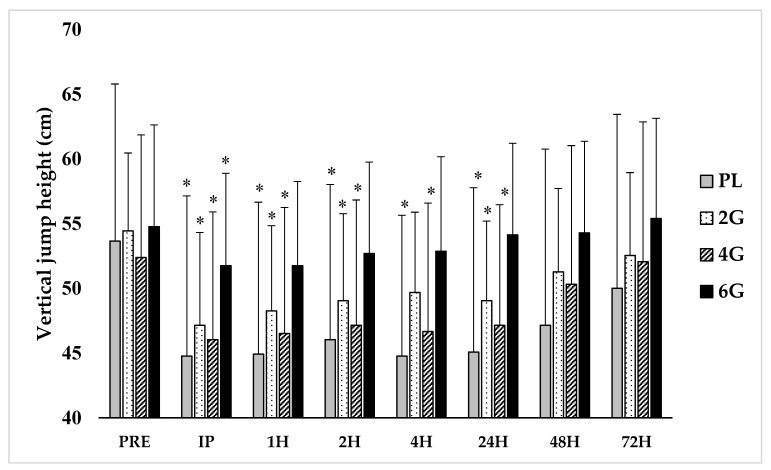
Effect of fish oil supplementation dosing on the recovery of vertical jump height following a bout of muscle-damaging exercise. (n = 32; n = 8 per group; data are presented as mean ± standard deviation)**.** * indicates statistical significance (*p* < 0.05) from PRE for a given fish oil dose; PRE = pre-exercise; IP = immediate post-exercise; H = hour post-exercise; PL = placebo; G = grams per day; cm = centimeters.

A large dosing effect was observed in the interaction between treatment and time on VJ height (F = 2.66, *p* < 0.001, η^2^ = 0.22). Post-hoc analysis indicated significant (*p* < 0.001) main effects for time for each group. At IP, VJ height was significantly reduced for PL (−17.4 ± 6.0%, *p* < 0.001), 2G (−13.9 ± 6.9%, *p* = 0.019), 4G (−12.4 ± 6.5%, *p* = 0.036), and 6G (−5.4 ± 2.8%, *p* = 0.022). However, VJ performance recovered to PRE by 1 h for 6G (*p* = 0.112), whereas it remained depressed (*p* < 0.03) from PRE for all other groups until 48H; an exception was noted for 2G at 4H (*p* = 0.13). No group differences were observed at any specific time point. The changes in VJ performance are illustrated in Figure 2.

Although repeated measures ANOVA did not reveal any other significant (treatment × time) interactions, a significant main effect for time was observed for MVIC (F = 43.68, *p* < 0.001, η^2^ = 0.61) and a tendency was noted for the 40-yd sprint time to improve (F = 3.26, *p* = 0.072, η^2^ =0.10) (Table 3 and Table 4). When the data were collapsed across groups, MVIC was significantly (all *p* < 0.001) reduced from PRE (234 ± 66 Nm) at IP (185 ± 65 Nm), 1H (181 ± 57 Nm), 2H (185 ± 58 Nm), 4H (188 ± 60 Nm), 24H (194 ± 65 Nm), 48 H (206 ± 66 Nm), and 72H (223 ± 70 Nm). Likewise, when the 40-yd sprint time data were collapsed across groups, significantly (*p* < 0.005) slower sprint times were observed at IP (6.20 ± 0.69 s), 1H (6.10 ± 0.65 s), 2H (6.07 ± 0.60 s), 4H (6.13 ± 0.71 s), 24H (6.13 ± 0.78 s), 48H (6.26 ± 2.15 s), and 72H (5.76 ± 0.59 s) compared to PRE times (5.63 ± 0.48 s). Complete data for MVIC and 40-yd sprint can be found in Table 3 and Table 4, respectively. No effects on T-test performance were observed.

### 3.2. Perceived Soreness

A large dosing effect was observed in the interaction between treatment and time on perceived soreness (F = 2.32, *p* < 0.001, η^2^ = 0.20). At IP, perceived soreness was similar (*p* > 0.05) to PRE-perceived soreness for all groups, though perceived soreness tended to be elevated for PL (4.5 ± 2.8 arbitrary units (au), *p* = 0.071) and 2G (3.4 ± 2.2 au, *p* = 0.093). Further, a difference (*p* = 0.024) was observed between 6G (1.5 ± 1.3 au) and 4G (5.5 ± 3.7 au). Subsequently, significant (*p* < 0.05) perceived soreness score elevations beginning at 1H and continuing through 48H were observed for PL and 4G; an exception was noted for PL at 4H (*p* = 0.147). For 2G, the perceived soreness score did not significantly rise until 24H (5.8 ± 1.7 au, *p* < 0.001), at which point it remained elevated through 48H (5.0 ± 1.6 au, *p* < 0.001).

**Figure 3:**
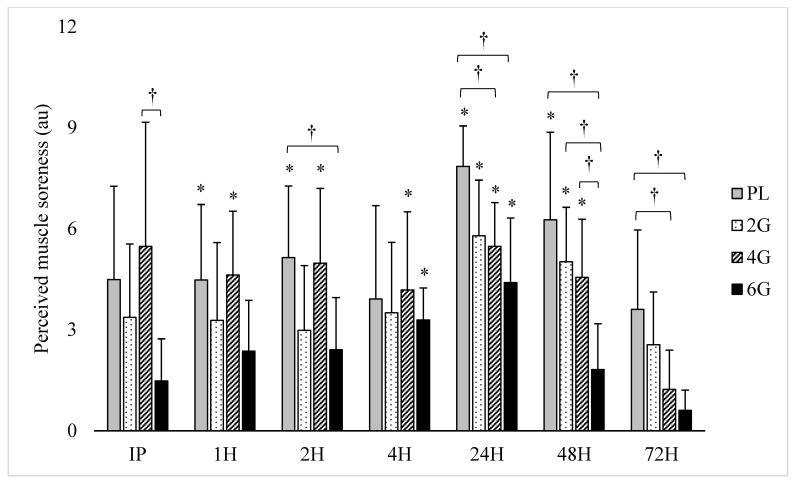
Effect of fish oil supplementation dosing on perceived muscle soreness following resistance exercise (mean ± SD). (n = 8/group; 4 males and 4 females per group). * Significant (*p* < 0.05) difference from PRE. † Significant (*p* < 0.05) difference between groups.

For 6G, the perceived soreness score was significantly elevated at 4H (3.3 ± 1.0 au, *p* < 0.001) and 24H (4.4 ± 1.9 au, *p* = 0.009). Lower perceived soreness scores were reported for 4G compared to PL at 24H (mean difference = 2.38, *p* = 0.023) and 72H (mean difference: 2.38, *p* = 0.025). Compared to 6G, greater perceived soreness scores were observed in PL at 2H (mean difference = 2.74, *p* = 0.046), 24H (mean difference: 3.45, *p* < 0.001), 48H (mean difference = 4.45, *p* < 0.001), and 72H (mean difference = 3.00, *p* = 0.003). Lower perceived soreness scores for 6G were also noted when compared to 2G (mean difference = 3.20, *p* = 0.011) and 4G (mean difference = 2.74, *p* = 0.034) at 48H. Other group differences were variable by time-point. The effect of fish oil dosing on perceived soreness is illustrated in Figure 3.

### 3.3. Indirect Markers of Muscle Damage

Group x time interactions were observed for CK (F = 2.63, *p* = 0.018, η^2^ = 0.22) and LDH (F = 4.00, *p* < 0.001, η^2^ = 0.30) (Table 5). Significant (*p* < 0.05) main effects for time were observed for all dosage groups in relation to PRE concentrations (CK and LDH). Compared to PL (CK: 1805 ± 2035 IU/L; LDH: 410 ± 200 IU/L), CK tended (*p* = 0.055) to be lower at 72H for 6G (114 ± 21 IU/L) while LDH tended to be lower for 6G at 24H (194 ± 49 IU/L) and 48H (198 ± 55 IU/L) before significantly (*p* = 0.005) lower concentrations were observed at 72H (131 ± 28 IU/L). At 24H, lower (*p* = 0.020) CK concentrations were observed in 6G (545 ± 151 IU/L) compared to 2G (3021 ± 1753 IU/L). At 48H, CK concentrations tended (*p* = 0.076) to be lower in 6G (261 ± 104 IU/L) compared to 4G (2188 ± 2110 IU/L). At 72H, lower (*p* = 0.005) LDH concentrations were observed in 6G compared to 2G (412 ± 135 IU/L).

## 4. Discussion

This is the first study to examine the dose–response impact of FO supplementation on markers of recovery following vigorous eccentric exercise. This is the second study with which our research group has utilized lower body eccentric resistance exercise to induce skeletal muscle damage in order to study recovery. Like a previous investigation [47], it appears from the significant time effects and magnitude of responses that the exercise protocol adequately induced skeletal muscle damage, as indirectly assessed by the post-exercise increases in participants’ CK and LDH concentrations, reductions in muscular performance, and increases in perceived soreness. Thus, we are confident that we were able to adequately examine the impact of different FO dosages on recovery.

To date, research on the role of FO on recovery from vigorous exercise has focused on a single dosage (2G for example) compared to a placebo, with the exception of Jakeman et al. [48], who used a single relative dose. With the previously published literature in mind, we aimed to explore 2-, 4-, and 6G per day of FO supplementation, as it spans many of the dosages studied to date. The primary finding from our research indicates that 6G of FO (2400 mg/day EPA and 1800 mg/day DHA) is most effective for delaying perceived muscle soreness following a damaging bout of exercise and perceived soreness at the 6G dose displayed lowered soreness ratings at all post-exercise time-points when compared to all other supplementation groups. Further, 6G enhanced the recovery of VJ performance, which was evident on the same day participants completed the damaging bout of eccentric exercise (at 1H post) and throughout the entire 72H recovery period. While statistical significance was variable, interestingly, 6G of FO tended to result in lower levels of markers of indirect muscle damage.

It is understood that the perception of soreness one experiences following unaccustomed or vigorous exercise results from increased nociceptor signaling, specifically group III and IV afferents [49,50]. More specifically, damaged skeletal muscle is infiltrated with inflammatory markers (e.g., proinflammatory prostaglandins, histamines, kinins) and edema, which apply pressure to the nociceptors [49,50,51]. Omega-3 fatty acids (i.e., EPA and DHA) found in FO supplements produce anti-inflammatory and inflammation-resolving mediators (e.g., protectins, resolvins, maresins), while simultaneously reducing the transcription of proinflammatory cytokine genes [52]. Thus, FO supplements have the potential to reduce the perception of soreness by indirectly decreasing nociceptor activation.

The literature to date has been published examining FO supplementation and perceived soreness following damaging or vigorous exercise [32,33,34,38,41,53,54,55,56]. While a direct comparison is impossible due to the array of damaging or vigorous exercise protocols that have been studied (e.g., eccentric squat, bench stepping, eccentric exercise of the elbow flexors), our finding that 6G FO supplementation reduces perceived soreness following a single bout of damaging exercise has been demonstrated in previous research. Specifically, our results are in agreement with an investigation by Tinsley et al. [33], who examined 6G of FO supplementation compared to PL. It was noted that females, who were untrained and consumed 6G FO, reported less muscle soreness at rest and during functional movements of both the upper and lower body following vigorous resistance exercise (10 sets to failure, 50% 1RM—both elbow flexion and knee extension). It should be noted that the findings were not statistically significant, but a 33% to 42% lower effect size for FO was noted in the post-exercise period. One prominent difference between the Tinsley et al. investigation and our study is that we employed a seven-week run-in the supplementation period prior to inducing muscle damage, while their experimental design included supplementation for seven days prior to the damaging exercise bout. To add, and in contrast to our findings, an investigation by McKinely-Barnard et al. [40] found that 6G per day FO for 21 days resulted in higher perceived soreness ratings by young women 6- and 24-H post-eccentric exercise when compared to PL. Interestingly, the investigation utilized the same supplementation (FO and PL brands) as our study. While our results support the higher dosage of 6G, the literature to date on this dose is variable.

Further, and importantly, previous research on FO dosages less than 6G has also yielded positive outcomes on the perception of soreness. An investigation of untrained men consuming 1.8G FO for 30 days demonstrated significant decreases in delayed onset muscle soreness at 48 h post-eccentric exercise (bench stepping) [57]. Similar results have also been noted by Jouris et al. [32] and Lembke and colleagues [35], who found 3G FO for 7 days and 2.7G FO for 30 days, respectively, resulted in significant decreases in perceived soreness. Overall, the majority of the literature to date tends to support supplementation with FO from low (1.8G) to higher (6G) dosages per day to decrease perceived soreness [32,33,35,38,53,54,56,57], though conflicting data do exist [34,41].

It has been highlighted that following the consumption of omega-3 fatty acids, omega-3 blood profiles increase, often in a dose-dependent manner, within days [58,59]. However, the incorporation of omega-3 fatty acids into skeletal muscle takes longer due to the slower fatty acid turnover in skeletal muscle [37,58]. As such, and to the best of our knowledge, the literature to date on FO supplementation and perception of soreness following vigorous training or skeletal muscle-damaging exercise ranges from 1 to 8 weeks in both trained and untrained men and women, with results indicating that both short and longer duration supplementation may positively impact the perception of soreness (i.e., lower it) following vigorous/damaging bouts of exercise (for a recent review, please see [22]). While our results positively support 7 weeks of supplementation with 6 g per day, compared with lesser dosages, we did not investigate shorter time periods, and thus more research is warranted.

While previous research has indicated FO supplementation may attenuate losses in muscular strength following vigorous exercise [35,54,55], our results do not support these findings, which is similar to the majority of research to date [34,40,41,53,55]. It is thought that FO may reduce the amount of damage assumed by the worked skeletal muscle and conserve strength and power production, and thus muscular performance. As such, we examined participants’ dominant knee extensors’ MVIC, as well as more applied aspects of muscular performance testing (T-test agility, 40-yd dash, and VJ). Our results indicated no meaningful impact of FO supplementation on MVIC, T-test agility, and 40-yd dash performance. Interestingly, 6G of FO supplementation positively impacted VJ recovery, an assessment of muscular power, at 24H post-exercise and throughout the remainder of the recovery period. As the VJ assessment is an indirect test of very brief lower-body power production [60], the simple decrease in perceived soreness in the 6G group may have contributed to these results. Interestingly, our VJ recovery results are similar to that of Jakeman and colleagues [60], despite the vast differences in FO supplementation time periods and different FO supplement regimens. Specifically, Jakeman et al. incorporated a single relative FO dose of either “high” (EPA 750 mg, DHA 50 mg) or “low” (EPA 150 mg, DHA 100 mg) at a dose of 1 g per 10 kg of body mass immediately post-plyometric exercise. Results from their investigation indicated that the decrement in VJ performance was ~4% after 1H for the high-EPA group in comparison with the ~10% and ~15% decrements observed for the control or low-EPA groups, respectively. The high-EPA group returned to within ~2% of the baseline performance after 24H, in comparison with the control and low-EPA groups, whereby their VJ performance was still ~4.4% and 3.8% below baseline at the 96H post-exercise time-point.

While our research is novel in that it is the first to examine the impact of different FO dosages with the same EPA/DHA ratio on recovery from skeletal muscle-damaging exercise, there are a few limitations to this research. We did not measure the increase in omega-3 fatty acid levels in the blood or skeletal muscle. Further, in females, we did not control for the menstrual cycle phase, which may have influenced our results. Lastly, our small sample size did not allow use to determine differences between sexes for dependent variables. This may have attributed to the lack of significant differences noted between groups, especially for blood enzymes. However, our research is the first to employ a comprehensive assessment of perceived soreness, muscular performance, and blood biomarkers following prolonged supplementation of varying FO doses in both men and women, making this research novel.

## 5. Conclusions

Based on the results of our investigation, we suggest exercising individuals undergoing vigorous or unaccustomed exercise consume a higher dose of 6G per day (2400 mg EPA, 1800 mg DHA) in order to reduce perceived soreness and improve acute power production in the recovery period.

The ideal dose of FO should continue to be researched in exercising men and women, with appropriate caution towards high-dose FO and the potential inhibition of platelet function [61]. Further, future investigations should consider incorporating a comprehensive assessment of inflammatory markers, as well as conducting acute and long-term FO supplementation periods at different dosages, as well as different EPA and DHA ratios. 

## Figures and Tables

**Table 1:** Participant descriptive for each supplement group. Participants were randomly assigned (n = 8/group; 4 males and 4 females per group) to their designated supplement group. No significant differences (*p* > 0.05), as determined via a one-way analysis of variance, were noted between groups for age, height, body mass, body fat percentage, or calorie or protein intake from day 42–day 52.

Participant Characteristics	2G	4G	6G	Placebo
Age (year)	23.5 ± 3.3	23.3 ± 3.0	23.8 ± 2.8	23.0 ± 3.0
Height (cm)	170.9 ± 6.9	172.9 ± 4.7	173.8 ± 7.6	173.6 ± 6.2
Body Mass (kg)	76.1 ± 14.2	69.7 ± 15.9	72.8 ± 13.5	67.9 ± 10.7
Body Fat (%)	20.8 ± 4.1	19.0 ± 6.2	19.4 ± 6.1	20.6 ± 7.2
Calorie Intake (kcal/day)-Familiarization-Experimental Trial (Day 42–Day 52)	2363.25 ± 489.13	2283.88 ± 375.98	2050.88 ± 552.04	2160.13 ± 415.21
Protein Intake (g/kg)-Familiarization-Experimental Trial (Day 42–Day 52)	1.28 ± 0.10	1.21 ± 0.24	1.23 ± 0.11	1.19 ± 0.17

**Table 2:** Eicosapentaenoic acid and docosahexaenoic acid totals per day of supplementation for each fish oil supplemented group.

Supplementation	EPA (mg)	DHA (mg)	Total EPA+DHA (mg)
2G FO	800	600	1400
4G FO	1600	1200	2800
6G FO	2400	1800	4200

FO = fish oil, G = grams per day, EPA = eicosapentaenoic acid, DHA = docosahexaenoic acid, mg = milligrams.

**Table 3:** Maximal voluntary isometric contraction of the dominant limb knee extensors for each group at all time-points. All values are newton-meters (Nm). n = 8/group; 4 males and 4 females per group

Time Point	2G	4G	6G	Placebo
PRE	233.26 ± 63.45	221.76 ± 50.21	250.74 ± 85.51	230.95 ± 70.36
IP	182.00 ± 63.79	172.55 ± 61.28	217.16 ± 70.46	169.64 ± 65.47
1H	182.41 ± 51.02	174.56 ± 54.36	196.23 ± 64.08	169.25 ± 65.89
2H	183.06 ± 53.45	174.85 ± 53.53	210.64 ± 68.17	172.95 ± 60.08
4H	189.03 ± 42.78	171.20 ± 56.02	222.51 ± 79.28	170.38 ± 62.35
24H	191.46 ± 57.54	178.75 ± 57.00	229.79 ± 74.16	175.64 ± 66.94
48H	204.10 ± 59.57	196.05 ± 65.85	231.13 ± 73.67	191.59 ± 68.22
72H	224.93 ± 62.66	205.76 ± 54.48	251.28 ± 89.69	211.43 ± 67.93

PRE = pre-exercise, IP = immediate post-exercise, H = hour, G = grams per day; data are presented as mean ± SD.

**Table 4:** Sprint (40-yd) time (s) for each group at all time-points. All values are seconds. n = 8/group; 4 males and 4 females per group

Time Point	2G	4G	6G	Placebo
PRE	5.69 ± 0.38	5.63 ± 0.55	5.51 ± 0.41	5.68 ± 0.61
IP	6.36 ± 0.53	6.17 ± 0.83	5.81 ± 0.31	6.46 ± 0.87
1H	6.25 ± 0.57	6.03 ± 0.64	5.74 ± 0.39	6.38 ± 0.84
2H	6.19 ± 0.49	6.04 ± 0.64	5.75 ± 0.45	6.31 ± 0.74
4H	6.19 ± 0.50	6.04 ± 0.61	5.73 ± 0.43	6.56 ± 1.02
24H	6.19 ± 0.57	6.13 ± 0.92	5.72 ± 0.31	6.48 ± 1.03
48H	5.93 ± 0.37	5.90 ± 0.70	5.68 ± 0.32	6.27 ± 0.92
72H	5.79 ± 0.44	5.79 ± 0.67	5.52 ± 0.41	5.96 ± 0.79

PRE = pre-exercise, IP = immediate post-exercise, H = hour, G = grams per day; data are presented as mean ± SD).

**Table 5:** Creatine kinase (CK) and lactate dehydrogenase (LDH) blood values for each group at all time-points. n = 8/group; 4 males and 4 females per group.

	Baseline	PRE	2-HR	4-HR	24-HR	48-HR	72-HR
Creatine Kinase (IU/L)							
PL	115.8 ± 70.5	116.1 ± 70.4	218.1 ± 67.9	355.8 ± 141.7	1751.9 ± 1397.3	1784.5 ± 1713.5	1804.9 ± 2034.8
2G	89.9 ± 44.1	89.6 ± 42.2	193.3 ± 93.9	427.6 ± 198.4	3020.6 ± 1753.4	2060.6 ± 1353.3	956.8 ± 692.7
4G	108.8 ± 59.2	101.9 ± 56.6	181.3 ± 72.4	435.8 ± 290.0	2058.0 ± 2217.7	2188.3 ± 2110.3	1371.1 ± 1309.9
6G	106.0 ± 17.0	102.9 ± 19.3	128.0 ± 58.8	285.1 ± 121.3	544.5 ± 150.8	261.1 ± 103.5	114.3 ± 21.1
Lactate Dehydrogenase (IU/L)							
PL	108.3 ± 52.6	109.5 ± 46.7	167.3 ± 74.7	193.9 ± 106.8	401.2 ± 165.3	423.3 ± 186.2	410.0 ± 200.3
2G	121.6 ± 16.5	121.4 ± 17.8	151.0 ± 39.9	188.6 ± 108.1	364.0 ± 139.7	397.5 ± 139.9	412.1 ± 135.0
4G	167.6 ± 87.5	149.6 ± 70.7	197.1 ± 73.2	210.3 ± 79.5	376.1 ± 212.5	375.9 ± 233.9	237.7 ± 180.1
6G	114.3 ± 15.6	114.5 ± 17.0	152.9 ± 45.5	181.4 ± 53.8	194.2 ± 49.2	198.0 ± 55.0	130.9 ± 28.3
